# Contextualising health screening risk assessments in police custody suites – qualitative evaluation from the HELP-PC study in London, UK

**DOI:** 10.1186/s12889-018-5271-6

**Published:** 2018-03-22

**Authors:** Iain McKinnon, Tracy Finch

**Affiliations:** 1 Institute of Neuroscience, Newcastle University, Newcastle upon Tyne, NE4 5PT UK; 2 Northumberland Tyne and Wear NHS Foundation Trust, Morpeth, NE61 3BP UK; 3 Department of Nursing, Midwifery and Health, Northumbria University, Coach Lane Campus West, Newcastle upon Tyne, NE7 7XA UK

**Keywords:** Police custody, Risk assessment, Health screening, Implementation, Normalization process theory, Criminal justice system

## Abstract

**Background:**

In the UK, police custody officers have a responsibility to screen for health morbidity and vulnerability among detainees. This study aimed to develop an understanding of the barriers to performing effective health screening in police custody suites, understand the impact of screening tools on practice within the custody suite, and to identify factors that could hinder or facilitate the future implementation of a new screening intervention in this environment.

**Methods:**

A qualitative study was conducted alongside a quantitative evaluation of a novel screening tool. Qualitative methods included observation of the custody environment, semi-structured interviews with police staff, and elicitation of comments from detainees about their experiences of screening. Data were analysed inductively using thematic analysis. Normalization Process Theory (NPT) was used to inform data collection and as a framework for higher level analysis of findings.

**Results:**

Five overall constructs were identified that develop understanding of the integration of health screening within custody: the workability of risk assessment screening tools; the effect of the custody environment and the people therein; shifts in professional roles and interrelationships amongst staff; cultural responses to risk and liability in police work; how infrastructure, knowledge and skills can impact on detainee safety.

**Conclusions:**

Health and risk assessment screening in police custody is a complex and demanding activity which extends beyond the delivery of a screening tool. Professional roles, the demanding environment and police culture impact on the overall process. Recommendations for improved integration of health and risk assessment screening in wider police custody practice are proposed.

**Electronic supplementary material:**

The online version of this article (10.1186/s12889-018-5271-6) contains supplementary material, which is available to authorized users.

## Background

Health morbidity among detainees in prisons worldwide has been well described [1, 2], and there is now a growing interest in the health and welfare of police custody detainees [3]. Emergent international literature highlights substance misuse & withdrawal, injuries, serious mental illness, suicidality and mental vulnerability amongst police detainees compared to general population estimates [4–8]. In addition, a number of studies have raised concerns about the ability of police custody officers to identify health needs, and calls for improvements in screening have been made [9, 10].

The treatment of police custody detainees varies from country to country, but in the UK any individual who is arrested must have their detention authorised by a police Custody Officer (CO). COs (sometimes known in the UK as desk sergeants) must independently scrutinise an arresting officer’s reasons for an arrest, and be satisfied that the conditions for detention are met. COs are also responsible for the identification of health morbidity and vulnerability among detainees and this is a key part of their safe detention. However COs as police officers are not clinically trained. Furthermore, there is evidence of shortcomings in routine screening (known by the police as “risk assessment”), especially for detainees with serious injuries, chronic physical health problems, mental disorders and vulnerability and those at risk of alcohol withdrawal [9, 11] with calls for the introduction of evidence based structured screening [12]. As discussed above, international studies show similar patterns of excess health morbidity, but the arrangements for the provision of healthcare in police settings, the process for how detainees are screened and access care, and the reporting of health outcomes vary substantially between nations [13].

From our Health Screening of People in Police Custody (HELP-PC) project in London UK, we previously published data from phases 1 and 2 which described deficiencies in the screening of physical, psychiatric and substance related disorders by COs [11, 14]. Phase 1 comprised an audit of custody records to ascertain the prevalence of morbidities documented by COs, and additionally to obtain a power calculation for phase 2. In phase 2, detainees underwent structured interviews by clinical researchers; the findings from these interviews were then compared to information recorded on the custody records to ascertain the sensitivity and specificity of the police risk assessment screen.

At that time, the Metropolitan Police Service in London used a computerised custody records system “NSPIS”^1^ which contained a risk assessment screening tool embedded into the program [15]. During phases 1 and 2, the NSPIS risk assessment being used (version MR09b) comprised 16 questions and 12 observations for the CO to assess the need for further intervention for the detainee. In phase 3, a new risk assessment screening tool was developed by the research team and was piloted in 2012. This is described in more detail in the methods section below. These three phases are henceforth referred to as the ‘quantitative evaluation’ of the HELP-PC project.

Among the deficiencies in standard custody risk assessment screening uncovered in phases 1 and 2 of the HELP-PC project were substantial numbers of missed cases of Diabetes Mellitus, cardiovascular disorders, Serious Mental Illness, intellectual and developmental disorders, risk of alcohol withdrawal syndrome, and detainees at high risk of suicide. The results of this pilot showed an improvement in the detection of key health morbidities, and these data are described elsewhere [16, 17].

Police custody suites are frenetic and chaotic environments, [18–20] and early experiences within phases 1 and 2 of the HELP-PC project identified that there was likely to be more to successful health screening and risk assessment than the ‘screening tool’ alone. Custody risk assessment takes place across UK jurisdictions, being a statutory task in England, Wales & Northern Ireland [21, 22] and as part of custody procedure in Scotland [23]. All detainees who have been arrested and detained in police custody are the responsibility of the COs. COs are required to screen each and every detainee to ascertain whether they are fit for detention, need to see a Health Care Professional (forensic physician or nurse) for a specific intervention relating to their physical or mental health, or require special assistance by an ‘Appropriate Adult’ when they undergo police interview due to their mental vulnerability [24]. COs are also responsible for the transfer of detainees to emergency departments should this be required. In UK practice, the healthcare provision in police custody suites has traditionally been provided by a forensic physician, usually contactable by telephone, and sometimes responsible for a number of custody suites. More recently, forensic nurses have started to assume these responsibilities, and are often present 24 h a day in the custody suite.

COs are expected to perform this task under significant pressure. During phases 1 and 2 of the HELP-PC project, researchers witnessed the conduct of risk assessment screening by COs being affected by factors such as the busy and unpredictable nature of events within the custody suite, and challenging and uncooperative behaviour from custody detainees. It was therefore important to understand how factors relating to the police staff, detainees, and events in custody may impact on standard screening processes and on the potential implementation of enhanced procedures in routine practice. To date, there is little published research that includes data from interviews with police custody staff, structured observations and the views of detainees regarding health screening in custody. Given the complexities identified in this sphere, implementation science can contribute to understanding the successful application of an intervention such as health screening or risk assessment, by providing a lens for investigating the implementation of screening that brings attention to factors affecting implementation ‘in practice’ [25].

Normalization Process Theory [NPT], as a recognised theoretical approach within the implementation science field, was applied during phase 3 of the study in parallel to the piloting of a new risk assessment screening tool in one London custody suite. NPT is a theory of implementation that explains the embedding and integration of a new practice within a setting, with reference to the individual and collaborative work required to achieve successful integration in practice [26, 27]. It comprises four constructs which assist in understanding the practicalities of making interventions work in everyday practice. A change in practice – and the tasks required of it – must make sense to the participants involved in the work (*coherence*). There must also be a commitment amongst participants to engage, both collectively and individually, in the work required of the intervention (*cognitive participation*). Participants must also be able to accomplish the work required of them (*collective action*). Collective action is achieved both in terms of how participants interact with the technology or intervention (workability) and through the relations amongst the different aspects of work involved (integration). Finally, there must be scope for participants to assess and appraise the effects of the intervention on their work and make modifications to the intervention or its organisation (*reflexive monitoring*) in order for a change in practice to become embedded. NPT thus provides a way of understanding why – and how – new practices become embedded, by focusing our attention on the work that is required to achieve it within the environment where the work is conducted.

The aim of this present study, which took place alongside the pilot of the new risk assessment screening tool [16], was to:

1. Develop an understanding of the barriers to performing effective screening;
2. Understand the impact of the screening tool on practice within the custody suite;
3. Identify factors that could hinder or facilitate the future implementation of a new screening intervention.

## Methods

### Context of the present study

This qualitative study was conducted alongside phase 3 of the HELP-PC quantitative evaluation which studied the sensitivity and specificity of a new health screening risk assessment conducted by COs in London, UK. The development of and quantitative evaluation of the new risk assessment screen has been described previously [16]. In summary, a new structured, interactive screening procedure using evidence based tools was developed by the research team, in conjunction with the police during 2011. It was then piloted by a team of 12 COs in one police custody suite in London between May and August 2012, with over 1200 detainees undergoing the new pilot risk assessment screen instead of the standard NSPIS process.

### Study design

Alongside the piloting phase of the new risk assessment screen in summer 2012, three key sources of qualitative data were obtained for analysis:

1. Observational field notes to capture events in the custody suite;
2. Confidential audio recorded semi-structured interviews with police custody staff (custody officers and forensic nurses);
3. Comments obtained from detainees during clinical research interviews.

Normalization Process Theory (NPT) was used to inform the interview topic guides and also as a focus for the field observations as well as providing a framework on which to reflect on the findings [26].

#### Researcher characteristics

At the time of this study, IM was a fully registered medical professional, a trainee in psychiatry, and was undertaking his National Institute for Health Research (NIHR) Doctoral Research Fellowship. He had also undertaken training in qualitative research, and elements of ethnography.

#### Field observations

Observational information in and around the custody suite was gathered; this included the observation of officers, other staff and non-staff participants, as well as reflections on notable scenarios. Examples of these included episodes where events in the custody suite impacted on COs carrying out their screening role, or where staff had informal conversations with researchers about issues relating to issues of detainees’ health and welfare. Observations took place over the course of the phase 3 new risk assessment screen pilot between 23 May and 17 August 2012, specifically during periods that the researcher was present in custody (52 days out of a total of 74 days’ of data collection related to the screening tool). As IM was already in the custody suite collecting data for the quantitative evaluation, opportunities for observations were taken at times when he was not interviewing detainees for that purpose. Therefore the opportunities to take a view of incidents unfolding or to have informal discussions with staff or detainees were not continuous. COs and forensic nurses in the core custody team were aware of IM’s involvement in the research project, but it was unfeasible to inform all potential attendees to the custody suite of his presence. Examples of these “passive participants” included detainees, solicitors, custody visitors, case investigation officers or members of police senior management. Disclosure of IM’s purpose and presence was limited to times when it was relevant to the particular information obtained (e.g. IM asked a staff member for permission to use details of an informal discussion), or when IM was asked about the nature and purpose of his work. In line with the unpredictable nature of the custody environment, an open approach to observation, rather than pre-selected activities or events, was taken in order to respond to events on the day. Observational aspects of data drew on facets of ethnography as consistent with the study objectives of understanding practices in context [28]. Data from these periods of observation were collected in anonymised field notes. Any descriptions of demeanour and observational clinical data made were intentionally non-specific; e.g. ‘appears intoxicated’, ‘difficulties understanding questions’, ‘behaviourally disturbed’. No identifiable data other than whether the person was an officer, staff member or detainee were recorded, although a description of a detainee’s age, gender or ethnicity was gathered where relevant. Where a participant divulged a notable comment for example when IM was observing the custody suite in general, or during an informal discussion with IM, this was followed up by respondent validation; IM informed the participant that he was in an observer role and that information specific or sensitive to a participant was used only following consent to use it in an anonymised form. Field notes were organised and cyphered by date.

#### Semi-Structured Interviews

Whilst the phase 3 HELP-PC pilot study was taking place, interviews with the end users of the custody risk assessment screen, the COs and forensic nurses, were conducted. All twelve COs and five forensic nurses who provided round the clock health input for detainees on site during the observational study were approached for consent to take part in a structured interview. This was to obtain their views on what factors impact upon health screening and risk assessment in the custody suite. COs and nurses had to find quiet periods where they could leave the custody suite for these interviews, as well as the researcher being free from carrying out interviews for the quantitative part of the HELP-PC study. Ultimately four COs and two forensic nurses were available and agreed to take part, although others informally indicated their willingness to be involved. It was hoped to recruit more of the staff members, but because IM was concurrently collecting data for the quantitative part of the study, time constraints were a particular factor. Interviews were semi-structured supported by a topic guide [29] which covered the interviewees’ views on risk assessment screening, what factors impact on its effectiveness, their views on improving matters and also their opinion about the standard and pilot screening tools. Interviews lasted between 20 min and 44 min, were audio recorded using digital recording equipment, transcribed verbatim, and stored securely on a password-protected hard drive. One of the interviews was interrupted after 22 min due to the room being booked by another officer, but it was immediately restarted and finished in an adjacent room. Interviews took place in available office space behind a closed door in the police station. These interviews were confidential and were not disclosed to anyone else. No repeat interviews were carried out. Interviewees were given an anonymous identifier as per their gender and professional role.

#### Detainees’ comments

The clinical research interview posed to detainees during the quantitative evaluation included a supplementary question *‘Do you have any comments about the way the police asked you about your health problems?’* This was in order to invite detainees’ views which would contribute to the overall qualitative data set. Responses were collated anonymously for analysis. Of the 323 detainees interviewed during the quantitative part of the project, 148 gave the researchers specific comments on this topic. Detainees’ comments varied; some replies were short although others gave more involved and thoughtful responses. These were documented verbatim and included for analysis with the observations and staff interviews, but because the main focus of the detainee interviews was the collection of medical data, time constraints and participant burden considerations precluded focused qualitative data collection with detainees. Although the structured comments lack relative depth, in this evaluation the inclusion of such data provided a breadth of understanding of the impact of the screening tool, particularly from the detainees’ perspectives. Comments made by individual detainees were coded commensurate with their study number in the quantitative evaluation.

### Data analysis

Data from the observational field notes, interviews with staff, and comments from detainees were analysed using the principles of constant comparative methodology [30]. Data from the three sources were coded separately using NVivo9 [31]. Codes were continually reviewed as analysis proceeded, and analysed for emergent themes. Emergent themes were then arranged under higher level themes. In the context of the higher level themes, and with the NPT framework in mind, all of the original codes were then reviewed again with reference to the original datasets. Where more appropriate emergent themes or higher level themes had emerged in the analysis for specific codes, datasets were reorganised accordingly. The consistency of interpretation of the codes emerging from the data was established by both authors independently coding two of the interview datasets, and discussing discrepant interpretations. The mapping of codes into subthemes and intermediate themes was performed jointly by both authors. In this paper, the emergent themes are presented in the results section, and NPT is used as a framework to reflect on the findings in the discussion.

## Results

The resultant arrangement of codes, emergent themes and higher level themes are included in supplementary files for each of the three datasets. These are:

- Additional file 1 - Framework of codes from detainee comments;
- Additional file 2 - Framework of codes from field notes;
- Additional file 3 - Framework of codes from semi-structured interviews with police staff

The coding and theme development is described in Table 1.

**Table 1:** Coding and theme development

Stage	Transaction	Outcome		
		Field observations	Interviews	Detainees’ comments
1	Three Datasets collated between May and August 2012 ↓	10 field observations over a three month period (May-Aug 2012)	6 interviews during August 2012	148 detainees’ comments over a three month period (May-Aug 2012)
2	Coding of all transcriptions/datasets ↓	20 codes obtained from field observations	186 Codes obtained from six interviews (four COs and two nurses)	24 codes obtained from detainee comments
3	First sorting of codes into emergent-themes ↓	12 emergent-themes identified	22 emergent-themes identified	7 emergent-themes
4	Higher-level themes developed from emergent-themes ↓	4 higher-level themes generated	5 higher-level themes generated	4 higher-level themes generated
5	Review of codes against transcript section – and reassignment of code to higher-level theme if necessary	This stage not applicable to this dataset	Six new emergent-themes identified - data eventually arranged into:5 higher level themes, 28 emergent-themes & 186 codes	This stage not applicable to this dataset
Codes: Emergent Themes: Higher Level Themes:		20 12 4	186 28 5	24 7 4

In total 13 higher level themes were generated from the three data sources. For the presentation of findings in this paper, these higher level themes were incorporated into five sets of issues for which the illustrative data is presented:

1. The workability of risk assessment screening tools;
2. The effect of the custody environment and the people therein;
3. Shifts in professional roles and interrelationships amongst staff;
4. Cultural responses to risk and liability in police work;
5. How infrastructure, knowledge and skills can impact on detainee safety.

The relationship between the 13 higher level themes and the five overall constructs is depicted pictorially in Additional file 4.

### Workability of the risk assessment screening tools

COs and forensic nurses expressed a range of views about the process of risk assessment screening, emphasising aspects such as time constraints, functionality, making it more user friendly and navigable (See Table 2). Participants were prompted to discuss risk assessment and screening in general terms. COs expressed a need for a screen with prompts and questions that were more relevant than those on the standard NSPIS screen (evaluated in phases 1 and 2), although they anticipated that this would take longer, and found that this was indeed the case with in the new risk assessment screen pilot phase (in phase 3). One CO expressed surprise that, until using the new screen, they had never been prompted to ask about serious health considerations such as head injuries or alcohol withdrawal.

**Table 2:** Workability of the screening tool and its items

FIELD OBSERVATIONS “Sergeants are getting a little quicker after just a few goes. Amazing what they pick up on that the study team hadn’t considered.” (Field note in custody para 2: 23 May 2012)	
STAFF INTERVIEWS “You can scrap (the old screening tool)…you can have people with potentially life threatening conditions who can walk through that…” (Male nurse 1) “You think ‘blimey they were leaving that to us weren’t they?’ We’re more informed now.” (Male CO 1) “Alcohol withdrawal, head injuries, medication… the [new] questionnaire highlights [them]. We never had [those previously].” (Male CO 3)	
DETAINEE COMMENTS “It was flippant. Very much a check list approach just like in supermarket.” (Male Detainee 338) “They’re very callous when asking questions as if to say ‘look, I’m reading you a list, just answer ‘no’ so I can get on.” (Male Detainee 29) “They’re getting into it a little bit more […]. It was a good thing.” (Male Detainee 234) “There was a lot more they could have asked. ‘Did you drop, did you fall? [’...] Sometimes you don’t know until later.” (Male Detainee 117) “Spent a lot of time on physical rather than mental illness.” (Male Detainee 311)	

Although these data were collected alongside the piloting of the new risk assessment screen in phase 3, staff inevitably commented on both the standard NSPIS and new screening processes. Staff experienced a range of challenges in working with both risk assessment screens. Some felt that both processes were perceived as ‘tick box’ to the detainees. Some COs thought in using the new screen, they were being asked to duplicate information being gathered by nurses. Other staff members, once familiarised with the new screen, became interested in reflecting on its strengths and relative weaknesses, giving suggestions on how to improve various aspects of it. Some detainees who were regular custody attenders noticed that the questions had changed since their last visit; some welcomed more detail, but others felt they were no better than before (Table 2).

### The effect of the custody environment and the people therein

Custody suites are busy, chaotic places, and the data revealed several ways in which the risk assessment tool did not fit well with the existing practices and demands of the custody environment (Table 3).

**Table 3:** Effect of the custody environment and the people therein

FIELD OBSERVATIONS “I spoke to the nurse regarding referrals to the local mental health services. He seemed rather vague about the referral criteria, and about what they would do when the local services would not see a detainee.” (Field note in custody para 4: 25 May 2012)	
STAFF INTERVIEWS “You could be dealing with a dozen active prisoners […] standing there staring at you, waiting [...] you just want to rush through the risk assessment just to get them out of the way. Time is the most crucial thing […] you can’t rush through it and tick lots of ‘YESes or NOs’ just by looking at them. Sometimes even the most straightforward question can take ages.” (Male CO2) “Ideally you should, in your formal handover, not only talk about the offence […] but also their risk assessment […] It should be formal, but generally, because it’s the end of your shift, you want to go.” (Male CO3) “Some people just will not cooperate no matter how much you’re trying to help them […] Others are so drunk [that] they don’t know where they are.” (Male CO2) “[Detainees] aren’t particularly forthcoming, even if it puts themselves at risk […] So you get people sitting in cells [who are] in life threatening situations” (Male nurse 1) “You’ve got constant communication with a nurse and people’s health can change rapidly. They [can] access to some information [or] previous records etcetera whilst we could be booking in the next person. They can actually say ‘do you know what Sarge, I think it might be this as well’ so you’ve got an ongoing continual assessment [with the nurse there]…it’s a massive difference.” (Male CO1) “One of the biggest issues we do have is people not being completely honest with us. If we got access for example to their GP records, we can verify [what they tell us] very quickly …” (Male nurse 1) “[Other places] have got mental health nurses. The mental health nurses decide whether to call out the crisis team or not. [Here] we don’t have mental health nurses; it’s either us or the Crisis Team.” (Female nurse 1) “Sometimes a custody suite is not the best place for an individual to be. There are probably other ways in which they could be dealt with. Not everybody needs to be arrested” (Female CO1)	
DETAINEE COMMENTS “They should ask you about your health in a more discreet fashion. They should check you into your cell and then ask you in private. People would talk more then.” (Female Detainee 202)	

The behaviour, attitudes, and intoxicated state of some detainees were seen by staff to impact upon the effectiveness of risk assessment. Staff were also concerned that the adversarial nature of the custody environment could dissuade detainees from disclosing important information about their health, such as mental health problems, or significant physical issues, and that consequently withholding this information could be detrimental to detainees’ welfare whilst in custody.

Lack of reliability of information from detainees increased the importance of professional lines of communication. Although COs saw the availability of permanent custody nurses as a positive factor in handling risk compared to the old system of a forensic physician on call, the appropriateness of managing some of the more serious health problems in custody was questioned by both professional groups. Access to General Practice records was suggested as advantageous. Handovers between officers’ shifts was also raised as an important vehicle to communicate important information about risk, but the lack of time meant that this information is not always effectively passed on.

Forensic nurses were frustrated by the lack of resources available to manage detainees with mental health conditions, especially the availability and responsiveness of community mental health services and crisis teams. It became apparent that some of the nurses were unclear about the protocols to obtain definitive assessments and management of acutely mentally unwell individuals.

These responses demonstrate the situated nature of risk assessment work within the challenging custody environment.

### Shifts in professional roles and interrelationships amongst staff

Using the new pilot health risk assessment screening tool raised questions about the relative roles and responsibilities of those involved in and beyond the custody environment (Table 4). Health screening of police custody detainees is a statutory requirement in England and Wales, and is the responsibility of the CO. There is an extended role for civilian staff called Detention Officers in performing the risk assessments, which has caused concerns for COs about their levels of individual responsibility for detainees’ health and wellbeing, especially where COs are being called upon to ‘supervise’ Detention Officers in taking on a role for which COs had legal responsibility.

**Table 4:** Shifts in professional roles and interrelationships amongst staff

STAFF INTERVIEWS “We are here to ensure that person’s safety and their wellbeing, regardless of what they have done. You have to detach yourself from what they have done.” (Female CO1) “Any medical screening should be done by a health care professional [and] with some sort of training. It shouldn’t be done by the custody sergeants. Some of them are very good and very knowledgeable, but I think that should be separate to the role” (Male nurse 1) “Would we have the time to do the risk assessments and see people? We can’t even get the nurses we need, let alone [more]” (Female nurse 1) “In [another police station] they have got 42 cells [and] they have two or three custody officers to oversee inputters’ risk assessments. You cannot monitor the behaviour of two detainees at the same time to make your judgement. Some poor Sergeant, hopefully not me, will be taken to task over it.” (Female CO1) “You’re just mindful of the new legislation [regarding] if someone dies in custody. If you haven’t done a half decent risk assessment you could be in a lot of trouble. You still have to write up, from your brief observations as they pass you, exactly what their issues are.” (Male CO2)	
DETAINEE COMMENTS “They were really concerned and help was provided.” (Male Detainee 333) “They should give up the questions. It’s too much to cope with when you first come in. [They should] get the doctor for everybody.” (Male Detainee 56)	

It was clear that COs sometimes experience a tension between the role of custodian and their welfare role. There were conflicting views between COs and forensic nurses regarding who should perform health screening. Some nurses thought that they should do this as a health care specialist, but recognised that currently limited resources would prevent it. Some COs felt that the way they managed risky detainees had changed over time especially in the light of changes to corporate manslaughter legislation. This was then seen as having become a much more defensive role than when some of the more seasoned officers first started working in custody.

Detainees held a range of views on the efforts of COs to look after their welfare. Some detainees found it difficult to understand that a uniformed officer’s duties could be consistent with looking after their safety. While some were sceptical about being screened by the CO, others had a better experience. Some detainees thought that a doctor or nurse should see all of the detainees, and that there was little to be gained by police officers asking health related questions.

### Cultural responses to risk and liability in police work

A key theme in the data revealed how policing culture impacted on the conduct of screening and risk assessment (Table 5). Participants’ comments reflected a perception that a ‘blame culture’ within the organisation filters down to COs, causing them to be risk averse. COs bring valuable professional experience to the screening process, but also draw on pre-formed ideas about mental health conditions when making judgements. Such ‘cognitive shortcuts’ may be taken for a number of purposes, such as reducing custody processing times or remaining in one’s skills comfort zone. The introduction of a structured and comprehensive screening tool thus challenges more instinctive practice.

**Table 5:** Cultural responses to risk and liability in police work

FIELD OBSERVATIONS “[I saw an] elderly detainee with Parkinson’s disease, on ropinirole medication [and an] indwelling urethral catheter [who had been] arrested on harassment charges. [The] nurse thinks that he has the catheter for the purpose of deriving sexual gratification [and] was unaware that ropinirole can cause sexual disinhibition. … [Nurse] continued to have the opinion that he was just a ‘nasty old man’.” (Field note in custody paras 1, 2 &3: 27 May 2012) “Detainees with multiple medical problems and a range of behaviourally disturbed presentations. Sergeants [were] keeping their cool. Voices were raised, and as a result the detainee’s voice is raised further and becomes more agitated. I suspect that a degree of frustration creeps in amongst the sergeants in these situations as they see a lot of this kind of presentation.” (Field note in custody para 1: 15 June 2012) “… [Detention officers] calling the detainee a ‘pain in the backside’. It is not difficult to sympathise with this point of view given the demands that are placed upon them by various detainees. However, it appears that they find it easy to ascribe behavioural disturbance to purely intrinsic factors but do not give consideration to any possible mitigating factors.” (Field note in custody para 1: 15 June 2012)	
STAFF INTERVIEWS “[The] thing that worries me most is missing something - so for instance going home with that ‘Oh God’ feeling” (Male CO1) “If they’re just mentally ill but they’re not having a crisis, they have to be dealt with as normal. I have been told in the past by the Crisis Team, ‘Well, we won’t come out; send them to prison – send them to court in the morning. Let them sort it out from there’.” (Female nurse 1) “People will always hurt themselves. I used to go, ‘Well more fool them.’ But I think, as I’ve had this role, you then go, ‘You’ve got a responsibility to look after them’.” (Male CO3) “Everything was covered in the pilot; it was just covered in too many questions. …I just feel that we have created…more work for ourselves where we’re taking less chances…some (custody officers) will not take a single chance… you’ve lost half your staff because they’re dealing with the constant watch.” (Male CO2)	
DETAINEE COMMENTS “Would like them to be more thorough about the state of mind. They should be more concerned. It’s like they don’t care.” (Male Detainee 2) “You see the nurse, tell them what the tablets are but you don’t get them. They don’t check on you.” (Female Detainee 284) “Didn’t like the way [the CO] judged me.’ You’re an alcoholic and crack and heroin addict.’ Think it messed my head up.” (Male Detainee 124)	

Detainees sometimes felt that they were not taken seriously by COs and nurses, and it was apparent that for some staff members, a sense of cynicism may have crept in after years of working in custody. One forensic nurse thought that the police had developed an ‘all or nothing’ view of mental health conditions that had developed from their experience that community mental health services would only respond to the most disturbed detainees, overlooking less poorly detainees who still have care needs.

There was a sense that COs felt under pressure to process detainees through custody as quickly as possible and that this may lead to them not paying enough attention to their health needs in difficult circumstances.

### How infrastructure, knowledge and skills can impact on detainee safety

Health screening in the custody environment requires particular forms of knowledge and skills, which varies amongst those involved in the process (Table 6). COs valued their own experiences in making health and welfare judgements, but had little formal training. Some staff felt that although training was important, it was ephemeral within the organisation. The personal qualities of the individuals involved are also a factor in determining the approach to the whole concept of looking after detainee welfare. There was clear evidence that some COs were able to use the information and exercise discretion where appropriate.

**Table 6:** How infrastructure, knowledge and skills can impact on detainee safety

FIELD OBSERVATIONS “The Sergeant… had seen a detainee over the weekend who had been caught removing items from a supermarket. I also saw the man who clearly had some form of cognitive impairment. He gave the man a verbal warning and contacted his sister to see if she could get him to see the GP. Earlier this morning, [the Sergeant] had tried to contact the sister again to see if she had managed to make any progress. It transpires that the sister had a stroke and died the previous day. The Sergeant and I had a discussion about this as he was clearly quite shocked. We talked about a number of things but the conversation culminated with us talking about how occasionally he sees people in custody who have real needs and that they can make a difference. He allowed me to use his exact words ‘We see so much s**t in here; we forget how to deal with the nice people’.” (Field note paras 1&2: 25 July 2012)	
STAFF INTERVIEWS “There is a custody course, about the people we deal with. But I think you learn so much by doing it rather than any course. We’ve had a death in custody and unfortunately it takes a death, sometimes for [serious issues] to come to the limelight.” (Male CO3) “The organisation takes a massive risk unless you have got someone trained sitting in a busy custody suite. But what Police Officers get in training is very minimal when it comes to mental health and why people behave in certain ways.” (Female CO1) “On some occasions, the police officer straight away says ‘Oh, and we’ve brought their insulin in with them,’ but that tends to be a very on the ball police officer [who] knows the nurse is going to ask for certain medications: the first thing they say to [the arrested person] is ‘Is there any medications you would like to bring in with you?’ Risk assessment should start way before they even get to the custody suite.” (Female nurse 1) “Not very many [custody nurses] have had mental health training. Most of them are from [emergency departments] and [medical admissions wards] and things like that.” (Female nurse 1) “I think that you learn to identify certain medications when people tell you they are on them. You learn to identify that certain medications are for certain ailments.” (Female CO1) “Given my experience, it was quite clear he was Asperger’s. I said to the [detention officers], ‘When you deal with him you’re going to have to give clear explanations.’ They said ‘… he was really odd with the fingerprint machine. He couldn’t cope with that at all’.” (Female nurse 1)	
DETAINEE COMMENTS “I told the police I’ve got asthma, I’d left [the inhaler) at home. They didn’t ask how severe the asthma was just how often do I get it. They did not offer to get me my pump or to get a pump for me.” (Male Detainee 34)	

Staff also thought that medical conditions and the need for medication should be considered before the detainee arrived in custody, which would require greater awareness among all officers, not just those working in custody.

## Discussion

The results reveal that there are many variables impacting on the capacity of staff working in a police custody suite to ensure that detainees’ health concerns are adequately managed. There are complex issues related to the people, processes, environment and cultural factors that exist in police custody settings, which impact on the conduct of health screening.

This study focused on the introduction of a new health risk assessment screen into police custody. It sought to identify factors affecting successful screening in general, how a new screening tool impacts on practice within the custody environment, and to inform how future implementation in routine practice might be hindered or facilitated. A range of data sources were used including direct observation of the custody suite, interviews with COs and forensic nurses, and detainee viewpoints.

There are a number of potential barriers to the effective conduct of health screening that were apparent from studying the use of the new screening tool in practice. From an NPT perspective, issues of workability which in this study refers to the ease of use of the tool in the custody context, were important. For example, there was resistance amongst COs to lengthening screening procedures due to perceived pressure to reduce custody processing times. Staff and detainees saw a busy environment and lack of privacy as an impediment to divulging important health issues, thus relational challenges were apparent. Situational factors distract COs from the assessment task, or put pressures on time for optimal screening. The range and acuity of morbidity in custody suites mirrors emergency departments [4, 32] yet COs are tasked with identifying these and taking appropriate action.

The implementation of the new screening tool had some observable impacts on practice within the custody environment. The concept of an improved screening tool had ‘made sense’ (held ‘coherence’ in NPT terms) on some levels - the existing NSPIS screening tool used by the police was seen as inadequate, lacking in opportunities to probe detainees further, and lacking the coverage of health issues required by the Police and Criminal Evidence Act (PACE) [21]. Some COs found the new risk assessment screen’s prompts and interactive ‘guidance’ helpful to steer their decision making. Similar computerised prompts have been shown to be useful in decision making in General Practice, [33] with thorough training to use decision aids improving end users’ confidence [34].

NPT provides a framework for understanding the ways in which implementing new practices can alter the relations amongst those involved, and their relative contributions to the work. The screening tool altered perceptions of roles and inter-professional relationships within and beyond the custody suite. Some police staff felt that professional boundaries among staff in the custody suite had become blurred, with COs being asked to pose more detailed screening questions akin to those used by forensic nurses. Equally nurses felt they were better placed to screen detainees for health and welfare considerations, although this was not their statutory role under PACE. Furthermore, civilian staff becoming screening ‘data-inputters’ received a mixed response from COs who were concerned about having to take personal responsibility for information gathered by less experienced staff. It is not possible to ascertain who is best placed to perform the risk assessment screen, but it is notable that COs expressed feeling more likely to document morbidity where there was a nurse readily available in the custody suite. In Tayside, one study found that strong collaborations between police officers and nurses strengthened the operational integrity of the service [35]. However data from this study suggests that some nurses may lack skills and confidence in some areas of health morbidity frequently encountered in police custody. COs emphasised the importance of their instincts and judgements, however, it remains unclear how much these positively affect decision making. Judgements are also influenced by COs’ and nurses’ assessments of the truthfulness of detainees’ accounts. Police staff perceived that detainees could be ‘hard to like’ whilst acknowledging that reason for arrest had to be set to one side. Underestimation of risk to health and safety among ‘hard to like’ individuals has been highlighted in psychiatric settings [36]. In an unpublished work, hypothetical risk scenarios were posed to eight COs, finding marked variations in their risk perceptions due to inadequacies of risk assessment tools, lack of training, and decision making based on *heuristic* processes [37]. From an NPT perspective, the embedding of the screening tool into routine practice requires attention to resolving issues of *relational integration* [38]. Having a standard screening tool, with a supportive process, thus appears important for all stakeholders in the health screening process [37].

The integration of the new risk assessment screen - and health morbidity screening generally - into wider police and custody processes is likely to be challenging. On an ideological level, a tendency towards risk aversion, individual liability and (as described by some participants) a ‘blame culture’ within policing, may inhibit wider implementation of improved screening processes. The identification of health problems or wellbeing concerns for detainees necessitates an appropriate response, and our study participants highlighted concerns about staff having the appropriate skills to deal with these issues. From a practical perspective, COs and nurses considered that difficulties accessing services, especially mental health care for detainees could have a significant bearing on how they answered the screening questions. In particular there was little awareness of, or ability to respond appropriately to the less clear cut cases of detainees with mental health conditions; embedded Criminal Justice Liaison and Diversion services were not in place during this study. It may be that their introduction into police custody can positively impact on police decision making and confidence, and although data regarding its effectiveness show promising results [39–42] this has yet to be fully established. Another example of difficulties with the availability of services once a problem is identified relates to Appropriate Adults (AA). Despite a statutory requirement to obtain AAs for “vulnerable detainees” [21] a recent report highlighted that the best predictor for AA use by the police was not actual need, but their availability, which varies greatly depending on local arrangements [24].

In this study, bureaucracy and a lack of perceived support from senior colleagues were highlighted as impacting on their ability to perform the custody role. Compatibility issues between systems and lack of adequate IT infrastructure, although not detailed in the results presented in this paper, were also apparent during this study and would need to be resolved for wider implementation [43].

Given the multitude of police roles, other pressures such as improving crime clear up rates may render forces reluctant to use scarce resources performing more detailed risk assessment screening, especially where they perceive they are trying to do nothing more than prevent rare events such as deaths in custody [44, 45]. Police were also concerned about increasing referrals to the forensic nurse or physician, but this was not borne out by the quantitative data which showed that referrals remained unaffected by using the pilot risk assessment screen [16].

Integration of the data from health screening with other police systems and health records was also raised in this study. In combination, an apparent lack of trust among detainees about the police handling their sensitive information, and a lack of access to detainees’ health records against which to triangulate their disclosures serves as a further barrier to confidence in screening. Integrating such records has proved difficult [46] although since this study all forensic nurses and physicians in London have access to the National Health Service (NHS) Summary Care Record (personal communication – Sergeant Cathy Nicholson via email 7 July 2016). Furthermore it is unclear whether integration between health and police records will facilitate trust between the two parties. Similar challenges in the integration of information across health and social care services have been identified in research using NPT to understand the lack of integration of telecare for chronic disease management [47]. This would be important to explore in further research in this context.

This study has also raised questions about when the process of risk assessment begins. COs felt that detainee screening should commence prior to detainees arriving in custody. This would appear to be a highly problematic concept given the issue of availability and delivery of appropriate training for all police officers, community support staff, and health care professionals revealed in this study [48]. It is therefore unclear how this might be optimally delivered. However during this study a structured Vulnerability Assessment Framework for the identification of mental health conditions by members of the public was being trialled by the Metropolitan Police Service (MPS) by officers ‘on the beat’ [49], and as a result this is now in place across London [50].

A limitation is that this is a relatively small qualitative study, although combining the observations, interview data and comments from detainees about their experiences provides a detailed understanding of stakeholders’ experiences of working with health and screening processes in this environment. There were only a small number of participants in appropriate positions from whom to sample for the formal interviews. It may be that there were other points of view among staff members who were not interviewed, thus raising the question of lack of data saturation, and limited external validity in relation to other custody settings nationally and internationally. However, efforts were undertaken to ensure good representation of available staff, including reminding police staff of the requests to take part. Furthermore, the main investigator (IM) was concurrently undertaking other research in the custody suite, which may have coloured participants’ responses to interviews, or changed staff behaviours knowing that there was a participant observer present. Asking detainees for only brief comments was pragmatic given they had already undergone a detailed clinical evaluation, but this will inevitably have caused an uneven balance in their contributions to this dataset relative to the police staff. More detailed evaluation of detainees view is therefore recommended in future work.

The contribution of observational data to this study is a strength. Given the time pressured nature of the environment, focused observation allowed us to improve the efficiency of interviews by shaping the focus of questioning towards key issues, and also presented opportunities to follow up emerging issues through informal conversations captured as part of the fieldwork. To date, appraisals of screening tools in custodial settings have been limited, and there have been no qualitative studies of this process. This study thus adds significantly to research in this field by providing an account of the human, organisational, professional and contextual factors that affect how screening procedures take place, which to our knowledge has not previously been described in this way.

Since these data were collected in 2012, there have been a number of developments to police custody healthcare in England and Wales. It was envisioned that the commissioning of healthcare would transfer to the NHS in 2016 as is the case in Scotland, but this did not take place [51]. There have also been moves to commission healthcare services, often outsourced from third party providers, with concerns raised about the standards of training of healthcare professionals [52], again in contrast to the situation in Scotland where national standards are established [53]. However, screening by COs still remains a statutory role in most UK jurisdictions, and the relationship between police officers and healthcare staff is of vital importance to ensure detainee health and safety, as highlighted within this study.

As mentioned above, the introduction of mental health Criminal Justice Liaison and Diversion Teams following the Bradley Report [54] means that there is now a wider access to mental health assessment and intervention in police custody suites. Future research relating to custody healthcare and risk assessment screening, should examine the role of these teams in effective screening and delivery of mental health interventions.

One police force in England (Northumbria Police) has now implemented the new “HELP-PC risk assessment tool” (since June 2016), with other police forces across the UK expressing an interest in it. Evaluation work with Northumbria Police is now underway, which will allow any further revisions to be made.

This study highlights the issues that need to be considered for optimal integration of health morbidity screening in custody, and the barriers that need to be addressed. In relation to Normalization Process Theory [26], the data from this study highlighted in some detail, issues concerning both ‘workability’ and ‘integration’ of the screening tool within practice on the custody suite, which are aspects of ‘collective action’ as presented with NPT. However, the data also indicated insights into wider implementation and integration of health screening within police work that if implemented more widely, are likely to raise important issues concerning boundaries between police work and ‘health’ work.

## Conclusions

This qualitative study shows that there is more to screening police custody detainees than simply delivering a screening tool. There needs to be consideration of the demanding environment in which this is performed, changing professional roles in custody, the influence of cultural aspects within the police and the need for more robust training for officers. It also needs to be considered whether every vulnerable detainee needs to be in police custody. Although improving the appropriateness and comprehensiveness of the risk assessment tools used in police custody has value, its utility will be diminished unless appropriate health services are available for detainees.

It is recommended that any future implementation of novel risk assessment screening tools in police custody, seeks to investigate these issues in more depth with an integrative mixed methods approach. This must adequately acknowledge the views of detainees, which should be central to any evaluation of the effectiveness of screening and risk assessment processes.

## Additional files

Additional file 1: Framework of codes from detainee comments. (DOCX 14 kb)

Additional file 2: Framework of codes from field notes. (DOCX 15 kb)

Additional file 3: Framework of codes from semi-structured interviews with police staff. (DOCX 40 kb)

Additional file 4: Relationship between higher level themes and overall constructs (DOCX 85 kb)

## Abbreviations

AA: Appropriate Adult
CO: Custody Officer (also known as Police Custody Sergeant)
HELP-PC: Health Screening of People in Police Custody project
MPS: Metropolitan Police Service
NHS: National Health Service (UK)
NPT: Normalization Process Theory
NSPIS: National Strategy for Police Information Systems (explanation in footnote)
PACE: Police and Criminal Evidence Act (1984)

## Footnotes

1 The National Strategy for Police Information Systems (NSPIS) custody management system was widely used by police forces in England and Wales for custody officers to record and store custody information. The detainee booking-in part of this system includes a health screen/risk assessment, which was the focus of the original evaluation in 2009–10.
