# Supplementary material for: Contextualising health screening risk assessments in police custody suites – qualitative evaluation from the HELP-PC study in London, UK
Source: BMC Public Health. 2018 Mar 22;18:393. doi: 10.1186/s12889-018-5271-6 (PMC5863839; doi:10.1186/s12889-018-5271-6)
Supplement: Supplementary file 1 — Framework of codes from detainee comments. (DOCX 14 kb) [file 12889_2018_5271_MOESM1_ESM.docx]

*Framework of codes from the detainee comments.*

| Higher-level themes | Emergent themes | Codes |
| --- | --- | --- |
| 1. Suggestions for improvement | Questions need to be improved | Difficult to understand some questions |
|  |  | Long winded process |
|  |  | Language issues |
|  |  | Need more on mental health |
|  |  | Needs to be more in depth |
|  |  | Should ask about communicable disease |
|  |  | Not thorough enough |
|  |  | Open ended questions of no use |
|  | Need to ask about health earlier | Need to ask about health before or at time of arrest |
| 2. Negativity regarding the police | Police not sufficiently engaged in the process | Felt that sgt judged him |
|  |  | Lack of concern by officers |
|  |  | Sgt not concerned enough |
|  |  | Sgts going through motions |
|  |  | Negative re attitudes of officers |
|  |  | Negative re sgts |
|  | Don’t trust the police to tell them health issues | Don't trust the police with health information |
|  |  | Everyone should see a HCP |
|  |  | Need more privacy and confidentiality |
|  | Cautious approach of Sgt | Sgts overly cautious |
| 3. Generally positive responses | Positive responses about the questions | Positive about screening questions |
|  | Positive responses about the sgt | Sgt did a good job |
| 4. Detainee factors influencing the answers to questions | Detainee may influence the outcome | Answers can be influenced by the person being asked them |
|  |  | Could make things up |
|  |  | Not in a fit state to answer properly so soon after arrest |
