# Supplementary material for: Contextualising health screening risk assessments in police custody suites – qualitative evaluation from the HELP-PC study in London, UK
Source: BMC Public Health. 2018 Mar 22;18:393. doi: 10.1186/s12889-018-5271-6 (PMC5863839; doi:10.1186/s12889-018-5271-6)
Supplement: Supplementary file 2 — Framework of codes from field notes. (DOCX 15 kb) [file 12889_2018_5271_MOESM2_ESM.docx]

*Framework of codes from fieldnotes.*

| Higher-level themes | Emergent themes | Codes |
| --- | --- | --- |
| 1. Custody environment | Influence of detainee factors | Detainee did not want to complete the whole interview |
|  | Challenges to carrying robust research and enquiry in police custody | Reflecting on the difficulty performing a project of this kind in custody |
|  | Problems with interagency working | Uncertainty regarding pathways for mentally disordered detainees. |
|  | Specialist opinions not readily available | Problems engaging with social services |
|  |  | Detainee able to “sail through” screening due to good social façade |
| 2. Education | Adequacy of training | Concern that one of the COs had “lost their confidence” because of the new screen although this was the opinion of the nurse. Possible interpersonal issues? |
|  |  | Skill mix in custody. |
|  | Good practice | CO was able to reflect on the diversity of people who come through the door and that discretion could be used to good measure. |
| 3. Culture in custody | Sense of blame culture | Does being watched have an impact |
|  | Change culture and management | Personal attributes contributing to the approach to new interventions |
|  |  | Varying interpretations of what is “fast” |
|  | Prejudiced and ingrained ideas | Do health care professionals working in challenging environments become assimilated into the attitudes of others in the same environment |
|  |  | Automatically ascribing behavioural disturbance to “a bad person” rather than considering all of the possibilities… is this any different to the public at large? |
|  | Adversarial standpoint | Ability of police staff to diffuse situations |
|  | Roles | Further evidence of role conflicts |
|  | Competing interests | Balance to be struck between what can be done and what should be done. Difficult to please everyone at all times. |

| 4. Processes | Issues around the acceptability of new technology | Spent extended time in custody. Possibly my own anxieties? |
| --- | --- | --- |
|  |  | Some users were taken by the more colourful interface (compared to NSPIS) |
|  |  | My own anxieties of wanting it to work and be accepted. |
|  |  |  |
|  | Technical Skills | Performance improved with familiarity |
