# Supplementary material for: Contextualising health screening risk assessments in police custody suites – qualitative evaluation from the HELP-PC study in London, UK
Source: BMC Public Health. 2018 Mar 22;18:393. doi: 10.1186/s12889-018-5271-6 (PMC5863839; doi:10.1186/s12889-018-5271-6)
Supplement: Supplementary file 3 — Framework of codes from semi-structured interviews with police staff. (DOCX 40 kb) [file 12889_2018_5271_MOESM3_ESM.docx]

*Framework of codes from the semi-structured interviews.*

| Higher-level themes | Emergent themes | | | | | | | | | | | | | | Codes |  |
| --- | --- | --- | --- | --- | --- | --- | --- | --- | --- | --- | --- | --- | --- | --- | --- | --- |
| 1. Roles | Recognising the importance of judgement and fears re: inputter role | | | | | | | | | | | | | | Sixth sense playing a role |  |
|  |  |  |  |  |  |  |  |  |  |  |  |  |  |  | Use of non-verbal cues |  |
|  |  |  |  |  |  |  |  |  |  |  |  |  |  |  | Importance of non-verbal cues |  |
|  |  |  |  |  |  |  |  |  |  |  |  |  |  |  | Escalation in risk taking when supervising multiple DDOs |  |
|  |  |  |  |  |  |  |  |  |  |  |  |  |  |  | Supervision of DDO inputters |  |
|  |  |  |  |  |  |  |  |  |  |  |  |  |  |  | Personal contact |  |
|  |  |  |  |  |  |  |  |  |  |  |  |  |  |  | Being upset – would others have realised the risk |  |
|  |  |  |  |  |  |  |  |  |  |  |  |  |  |  | DDO inputters with little experience |  |
|  | Conflicts between sergeants’ statutory role and the HCP role: boundaries | | | | | | | | | | | | | | Health screening role should be done by the HCP |  |
|  |  |  |  |  |  |  |  |  |  |  |  |  |  |  | HCP role is advisory – Sgt holds the responsibility |  |
|  |  |  |  |  |  |  |  |  |  |  |  |  |  |  | Remains a need for triage |  |
|  |  |  |  |  |  |  |  |  |  |  |  |  |  |  | RA can assist in the decision making around rights and entitlements |  |
|  |  |  |  |  |  |  |  |  |  |  |  |  |  |  | Identification of health problems |  |
|  |  |  |  |  |  |  |  |  |  |  |  |  |  |  | Fear of having core roles eroded or altered |  |
|  | Tension between the welfare role and the custodial role | | | | | | | | | | | | | | Detainees don’t understand the Sgts’ welfare role fully |  |
|  |  |  |  |  |  |  |  |  |  |  |  |  |  |  | Detainee welfare |  |
|  |  |  |  |  |  |  |  |  |  |  |  |  |  |  | Detach detainee from the offence |  |
|  |  |  |  |  |  |  |  |  |  |  |  |  |  |  | Confidentiality |  |
|  |  |  |  |  |  |  |  |  |  |  |  |  |  |  | Is detainee’s attitude linked to the Sgts attitude |  |
|  | How the CNP is seen as part of the MPS | | | | | | | | | | | | | | Seen as an adversarial process |  |
|  | Team dynamics | | | | | | | | | | | | | | Potential for dysfunctional dynamics within the team |  |
|  |  |  |  |  |  |  |  |  |  |  |  |  |  |  | Arresting officers can worsen the behaviour of the detainee |  |
|  |  |  |  |  |  |  |  |  |  |  |  |  |  |  | Mutual trust |  |
|  |  |  |  |  |  |  |  |  |  |  |  |  |  |  | Need for a joined up approach |  |
|  |  |  |  |  |  |  |  |  |  |  |  |  |  |  | Sense of belonging to a team |  |
|  | Experience of stakeholders/staff within custody | | | | | | | | | | | | | | Little mental health experience among custody CNPs |  |
|  |  |  |  |  |  |  |  |  |  |  |  |  |  |  | Sgts know when to take risk management measures |  |
|  |  |  |  |  |  |  |  |  |  |  |  |  |  |  | Experience of the person undertaking the risk assessment |  |
|  |  |  |  |  |  |  |  |  |  |  |  |  |  |  | Cynicism about veracity of detainees report as a result of length of time performing role |  |
|  |  |  |  |  |  |  |  |  |  |  |  |  |  |  | Could MH nurses deal with high risk physical problems? |  |
|  | | | | |  | | | | | | | | |  | |  |
| 2. Screening tools/ Technology | | | | | Pilot screening tool issues | | | | | | | | | Pilot highlighting new areas | |  |
|  |  |  |  |  |  |  |  |  |  |  |  |  |  | Value of more prompts in the pilot risk assessment | |  |
|  |  |  |  |  |  |  |  |  |  |  |  |  |  | Issues with linkage to suicide section | |  |
|  |  |  |  |  |  |  |  |  |  |  |  |  |  | Concerns about increased length | |  |
|  |  |  |  |  |  |  |  |  |  |  |  |  |  | Perception of unacceptable length based upon first impressions | |  |
|  |  |  |  |  |  |  |  |  |  |  |  |  |  | Pilot too long | |  |
|  |  |  |  |  |  |  |  |  |  |  |  |  |  | Repetition | |  |
|  |  |  |  |  |  |  |  |  |  |  |  |  |  | Longer tool can lead sgts to lose interest | |  |
|  |  |  |  |  |  |  |  |  |  |  |  |  |  | Ambiguity in the pilot questions | |  |
|  |  |  |  |  |  |  |  |  |  |  |  |  |  | Slight change in stance re the relative downside of length | |  |
|  |  |  |  |  |  |  |  |  |  |  |  |  |  | Increased workload | |  |
|  |  |  |  |  | NSPIS screening tool issues | | | | | | | | | Deficiencies in current risk assessment process | |  |
|  |  |  |  |  |  |  |  |  |  |  |  |  |  | Current risk assessment questions are not specific enough | |  |
|  |  |  |  |  |  |  |  |  |  |  |  |  |  | Current risk assessment not effective enough | |  |
|  |  |  |  |  |  |  |  |  |  |  |  |  |  | Poor questionnaire design | |  |
|  |  |  |  |  | Procedure vs judgement | | | | | | | | | Balancing act between simplicity and sufficient detail | |  |
|  |  |  |  |  |  |  |  |  |  |  |  |  |  | Discretion in what to ask | |  |
|  |  |  |  |  |  |  |  |  |  |  |  |  |  | Person doing risk assessment needs to probe more than the standard questions | |  |
|  |  |  |  |  |  |  |  |  |  |  |  |  |  | Tension between being open and being directive | |  |
|  |  |  |  |  |  |  |  |  |  |  |  |  |  | Need for a nonstandard approach at times | |  |
|  |  |  |  |  |  |  |  |  |  |  |  |  |  | More to risk assessment than just the norm | |  |
|  |  |  |  |  |  |  |  |  |  |  |  |  |  | Structured observational cues versus intuition | |  |
|  |  |  |  |  |  |  |  |  |  |  |  |  |  | Tension between making experiential judgements and being forced to use a structured tool | |  |
|  |  |  |  |  |  |  |  |  |  |  |  |  |  | Do sergeants read and interpret questions appropriately | |  |
|  |  |  |  |  |  |  |  |  |  |  |  |  |  | Need to be able to record observations and justifications | |  |
|  | | | | Not static | | | | | | | | | RA is a dynamic process | | |  |
|  |  |  |  |  |  |  |  |  |  |  |  |  | Risk assessment is dynamic | | |  |
|  |  |  |  | Exceptions | | | | | | | | | With autism there is no easy answers and it is beyond a simple screening tool | | |  |
|  |  |  |  | IT | | | | | | | | | System glitches | | |  |
|  |  |  |  |  |  |  |  |  |  |  |  |  | IT issues are a problem | | |  |
|  |  |  |  | Impact | | | | | | | | | Sense that pilot is picking up more | | |  |
|  |  |  |  |  |  |  |  |  |  |  |  |  | Felt better informed to perform risk assessment | | |  |
|  |  |  |  |  |  |  |  |  |  |  |  |  | Sense that the pilot may have changed practice | | |  |
|  |  |  |  |  |  |  |  |  |  |  |  |  | Educational aspect to the pilot – might change practice | | |  |
|  |  |  |  | Wish list for a screening tool | | | | | | | | | Focussing on the important issues | | |  |
|  |  |  |  |  |  |  |  |  |  |  |  |  | Questions are personal in nature | | |  |
|  |  |  |  |  |  |  |  |  |  |  |  |  | Need for updates in keeping with emerging evidence | | |  |
|  |  |  |  |  |  |  |  |  |  |  |  |  | User dependent | | |  |
|  |  |  |  |  |  |  |  |  |  |  |  |  | Need for easy aide-memoires | | |  |
|  |  |  |  |  |  |  |  |  |  |  |  |  | Ordering of questions | | |  |
|  |  |  |  |  |  |  |  |  |  |  |  |  | Cultural insensitivities of basic screening questions | | |  |
|  |  |  |  |  |  |  |  |  |  |  |  |  | Need for comprehensive questions to be visible | | |  |
|  |  |  |  |  |  |  |  |  |  |  |  |  | Risk that people answer yes when prompted | | |  |
|  |  |  |  |  |  |  |  |  |  |  |  |  | Asking questions may prompt people to answer yes to something trivial | | |  |
|  |  |  |  |  |  |  |  |  |  |  |  |  | Interactivity is helpful | | |  |
|  |  |  |  |  |  |  |  |  |  |  |  |  | Need for user friendly format | | |  |
|  |  |  |  |  |  |  |  |  |  |  |  |  | Need for streamlined interface I’d like an improvement in identifying the person in front of me | | |  |
|  | | |  | | | | | |  | | | | | | | |
| 3. Culture of the institution | | | Fear of getting it wrong | | | | | | Risk aversion of some sgts | | | | | | | |
|  |  |  |  |  |  |  |  |  | Sharing risk or passing buck | | | | | | | |
|  |  |  |  |  |  |  |  |  | Defensive standpoint | | | | | | | |
|  |  |  |  |  |  |  |  |  | Personal experiences | | | | | | | |
|  |  |  |  |  |  |  |  |  | Dealing with people’s lives | | | | | | | |
|  |  |  |  |  |  |  |  |  | Cautious approach to risk assessment | | | | | | | |
|  |  |  |  |  |  |  |  |  | Fear of missing something significant | | | | | | | |
|  |  |  | Stigma surrounding MH and associated disorders | | | | | | Polarisation of mental health – all or nothing | | | | | | | |
|  |  |  |  |  |  |  |  |  | Need to recognise link between reducing MH problems and reducing offending | | | | | | | |
|  |  |  |  |  |  |  |  |  | Sgt senses link between mental disorders and deceit | | | | | | | |
|  |  |  | Pre-conceived ideas | | | | | | Things aren’t always what they seem at first sight | | | | | | | |
|  |  |  |  |  |  |  |  |  | Despondency about what can be done | | | | | | | |
|  |  |  |  |  |  |  |  |  | Are preconceived ideas held despite data showing otherwise | | | | | | | |
|  |  |  |  |  |  |  |  |  | Custody is cheaper than MH services for the mentally ill | | | | | | | |
|  |  |  |  |  |  |  |  |  | Predictability about what some Sgts will input | | | | | | | |
|  |  |  | Staff behaviour and attitudes | | | | | | Experience has changed approach to welfare issues | | | | | | | |
|  |  |  |  |  |  |  |  |  | Some do the bare minimum | | | | | | | |
|  |  |  |  |  |  |  |  |  | PNC checks will be done. Is this the minimum? | | | | | | | |
|  |  |  |  |  |  |  |  |  | In a comfort zone – not wanting to be challenged | | | | | | | |
|  |  |  |  |  |  |  |  |  | Join up arrest and interview for certain detainees | | | | | | | |
|  | | Culture of policing | | | | | | Not everyone needs to be arrested | | | | | | | | |
|  |  |  |  |  |  |  |  | Link between perceived ‘blame culture’ and increased awareness | | | | | | | | |
|  |  |  |  |  |  |  |  | Is the risk assessment seen as less important than other issues | | | | | | | | |
|  |  |  |  |  |  |  |  | Organisational shortcuts | | | | | | | | |
|  |  |  |  |  |  |  |  | Little supervision or mentorship for HCPs | | | | | | | | |
|  |  |  |  |  |  |  |  | Sense of financially driven motivation for changes | | | | | | | | |
|  |  |  |  |  |  |  |  | Getting more from less resource | | | | | | | | |
|  |  |  |  |  |  |  |  | Is CCTV a false friend? | | | | | | | | |
|  |  |  |  |  |  |  |  | Procedural drift – herd mentality | | | | | | | | |
|  | | | | | | |  | | | |  | | | | | |
| 4. Environment | | | | | | | Detainee behaviour | | | | Aggravating factors | | | | | |
|  |  |  |  |  |  |  |  |  |  |  | Detainee dependent | | | | | |
|  |  |  |  |  |  |  |  |  |  |  | Constant changes in the detainee’s demeanour and state of mind | | | | | |
|  |  |  |  |  |  |  |  |  |  |  | Detainees lose interest | | | | | |
|  |  |  |  |  |  |  |  |  |  |  | Detainees cannot concentrate | | | | | |
|  |  |  |  |  |  |  |  |  |  |  | Need to cross check names but can be difficult when straight to cell etc. | | | | | |
|  |  |  |  |  |  |  |  |  |  |  | “They don’t know and then of course if flags up enhanced risks and everything. It transpires that they haven’t taken their medication in two years, but because they’re in custody they need it.” | | | | | |
|  |  |  |  |  |  |  |  |  |  |  | Some detainees know what to say | | | | | |
|  |  |  |  |  |  |  |  |  |  |  | Behaviour at arrest seen as being as a result of hating the police. | | | | | |
|  |  |  |  |  |  |  |  |  |  |  | Substances and behaviour | | | | | |
|  |  |  |  |  |  |  |  |  |  |  | Accuracy of detainee self-report | | | | | |
|  |  |  |  |  |  |  |  |  |  |  | Impact of drugs | | | | | |
|  |  |  |  |  |  |  |  |  |  |  | Aggressive detainees | | | | | |
|  |  |  |  |  |  |  |  |  |  |  | Some may be less inclined to ask for help | | | | | |
|  |  |  |  |  |  |  |  |  |  |  | Do detainees know that they disclose and the implications | | | | | |
|  |  |  |  |  |  |  |  |  |  |  | Disparities in reports between Sgt and HCP | | | | | |
|  |  |  |  |  |  |  |  |  |  |  | Disparity in disclosures between Sgt and HCP | | | | | |
|  |  |  |  |  |  |  |  |  |  |  | Detainees don’t fully understand the ramifications of not cooperating with the risk assessment. | | | | | |
|  |  |  |  |  |  |  |  |  |  |  | Inevitability in some cases | | | | | |
|  | | | | | | Resources | | | | Value of the backup afforded by having permanent custody nurses available | | | | | | |
|  |  |  |  |  |  |  |  |  |  | Appropriate use of resources | | | | | | |
|  |  |  |  |  |  |  |  |  |  | Access to another opinion | | | | | | |
|  |  |  |  |  |  |  |  |  |  | Access to existing medical records | | | | | | |
|  |  |  |  |  |  |  |  |  |  | Benefits of permanent mental health teams being available | | | | | | |
|  |  |  |  |  |  |  |  |  |  | Could make better use of available resources | | | | | | |
|  |  |  |  |  |  |  |  |  |  | Reassurance of having full-time HCP on site | | | | | | |
|  |  |  |  |  |  |  |  |  |  | Disparities about nursing cover | | | | | | |
|  |  |  |  |  |  |  |  |  |  | Benefits of having permanent custody staff | | | | | | |
|  |  |  |  |  |  |  |  |  |  | Confounding effect of permanent CNP on referral rates | | | | | | |
|  |  |  |  |  |  |  |  |  |  | Financial considerations outweighing clinical need | | | | | | |
|  |  |  |  |  |  |  |  |  |  | Differences between FME and CNP in terms of the way sergeants can communicate | | | | | | |
|  |  |  |  |  |  | Busy | | | | Limited capacity in the suite when busy | | | | | | |
|  |  |  |  |  |  |  |  |  |  | Complex and busy environment | | | | | | |
|  |  |  |  |  |  |  |  |  |  | Busy custody suite | | | | | | |
|  |  |  |  |  |  |  |  |  |  | Difficulties attending fully to the task | | | | | | |
|  |  |  |  |  |  |  |  |  |  | Multiple distractions in play | | | | | | |
|  |  |  |  |  |  |  |  |  |  | Time pressures | | | | | | |
|  |  |  |  |  |  |  |  |  |  | Need to concentrate on RA | | | | | | |
|  |  |  |  |  |  |  |  |  |  | Sgts are able to filter out background noise | | | | | | |
|  |  |  |  |  |  |  |  |  |  | Competing interests in the booking process (or general process) RA culture and tool | | | | | | |
|  |  |  |  |  |  |  |  |  |  | Time consuming looking at prior risk assessments. | | | | | | |
|  |  |  |  |  |  |  |  |  |  | Importance of handover – felt to be rushed | | | | | | |
|  |  |  |  |  |  |  |  |  |  | Some sergeants don’t bother if it is obvious | | | | | | |
|  | | | | | | Setup of the suite | | | | Variations between police custody suites | | | | | | |
|  |  |  |  |  |  |  |  |  |  | More privacy would be desirable | | | | | | |
|  |  |  |  |  |  |  |  |  |  | Environment unconducive to disclosure of personal information | | | | | | |
|  |  |  |  |  |  | Managing health disorder in custody | | | | Positional asphyxia risk | | | | | | |
|  |  |  |  |  |  |  |  |  |  | Range of problems seen | | | | | | |
|  |  |  |  |  |  |  |  |  |  | Rapidly changing clinical presentation | | | | | | |
|  |  |  |  |  |  |  |  |  |  | Frustration with service offered by outside agencies | | | | | | |
|  |  |  |  |  |  |  |  |  |  | Dealing with health problems that shouldn’t be in custody | | | | | | |
|  |  |  |  |  |  |  |  |  |  | Dealing with many individuals who may not have GPs or UK medical records | | | | | | |
|  |  |  |  |  |  |  |  |  |  | Patient safety – access to medical records | | | | | | |
|  |  |  |  |  |  |  |  |  |  | Need to triangulate all available information | | | | | | |
|  | | | | | |  | | | | | |  | | | | |
| 5. Education/ Training | | | | | | Sgt / staff understanding of phenomen-ology of mental disorder | | | | | | Conceptual confusion in the link between mental disorder and crime | | | | |
|  |  |  |  |  |  |  |  |  |  |  |  | Perception of need – difference between nurse and crisis team (mental health) | | | | |
|  |  |  |  |  |  |  |  |  |  |  |  | Is link between cannabis and psychosis important? | | | | |
|  |  |  |  |  |  |  |  |  |  |  |  | When does mental disorder preclude culpability? | | | | |
|  |  |  |  |  |  |  |  |  |  |  |  | Need to recognise degrees of disorder | | | | |
|  |  |  |  |  |  |  |  |  |  |  |  | Complex co-existence of MH problems and substances | | | | |
|  |  |  |  |  |  |  |  |  |  |  |  | Even obviously severe cases can be pursued via CJS rather than MH services | | | | |
|  |  |  |  |  |  |  |  |  |  |  |  | Some cases where you will never foresee the risk | | | | |
|  |  |  |  |  |  |  |  |  |  |  |  | Different interpretations of what requires an appropriate adult | | | | |
|  |  |  |  |  |  |  |  |  |  |  |  | The traditional view of what requires an AA needs to be challenged | | | | |
|  |  |  |  |  |  | Sgt levels of clinical knowledge | | | | | | Lack of understanding of physiological mechanisms | | | | |
|  |  |  |  |  |  |  |  |  |  |  |  | More interventional approach | | | | |
|  |  |  |  |  |  |  |  |  |  |  |  | Simplistic approach to risk | | | | |
|  |  |  |  |  |  |  |  |  |  |  |  | Some health problems will always get through | | | | |
|  |  |  |  |  |  |  |  |  |  |  |  | Using knowledge to make sense of info detainee gives | | | | |
|  |  |  |  |  |  | Process of risk assessment | | | | | | Risk assessment should start prior to arrest | | | | |
|  |  |  |  |  |  |  |  |  |  |  |  | Setting the scene on arrival | | | | |
|  |  |  |  |  |  |  |  |  |  |  |  | Building rapport | | | | |
|  |  |  |  |  |  |  |  |  |  |  |  | Need to consider medical conditions and medication at the point of arrest | | | | |
|  |  |  |  |  |  | Formal / informal training | | | | | | Learning on the hoof | | | | |
|  |  |  |  |  |  |  |  |  |  |  |  | Need for appropriate training | | | | |
|  |  |  |  |  |  |  |  |  |  |  |  | Little mental health training for CNPs | | | | |
|  |  |  |  |  |  |  |  |  |  |  |  | Training needs to extend to officers in general | | | | |
|  |  |  |  |  |  |  |  |  |  |  |  | Training is reactive not proactive | | | | |
|  |  |  |  |  |  |  |  |  |  |  |  | Lack of training | | | | |
