# Supplementary figures and images for: Contextualising health screening risk assessments in police custody suites – qualitative evaluation from the HELP-PC study in London, UK
Source: BMC Public Health. 2018 Mar 22;18:393. doi: 10.1186/s12889-018-5271-6 (PMC5863839; doi:10.1186/s12889-018-5271-6)

Supplementary figure - Relationship between higher level themes and overall constructs.docx


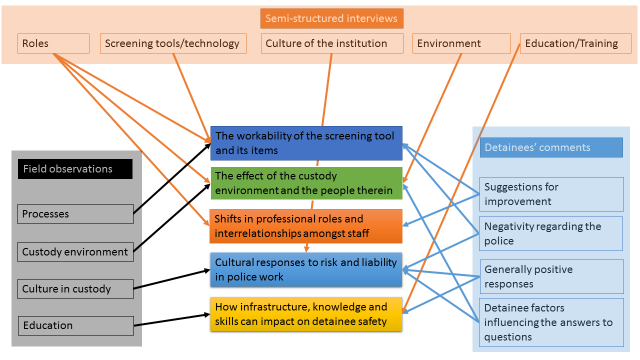

Supplement: Supplementary file 4 — Relationship between higher level themes and overall constructs (DOCX 85 kb) [file 12889_2018_5271_MOESM4_ESM.docx]
